# Supplementary figures and images for: Ripening dynamics revisited: an automated method to track the development of asynchronous berries on time-lapse images
Source: Plant Methods. 2023 Dec 14;19:146. doi: 10.1186/s13007-023-01125-8 (PMC10720176; doi:10.1186/s13007-023-01125-8)

**A**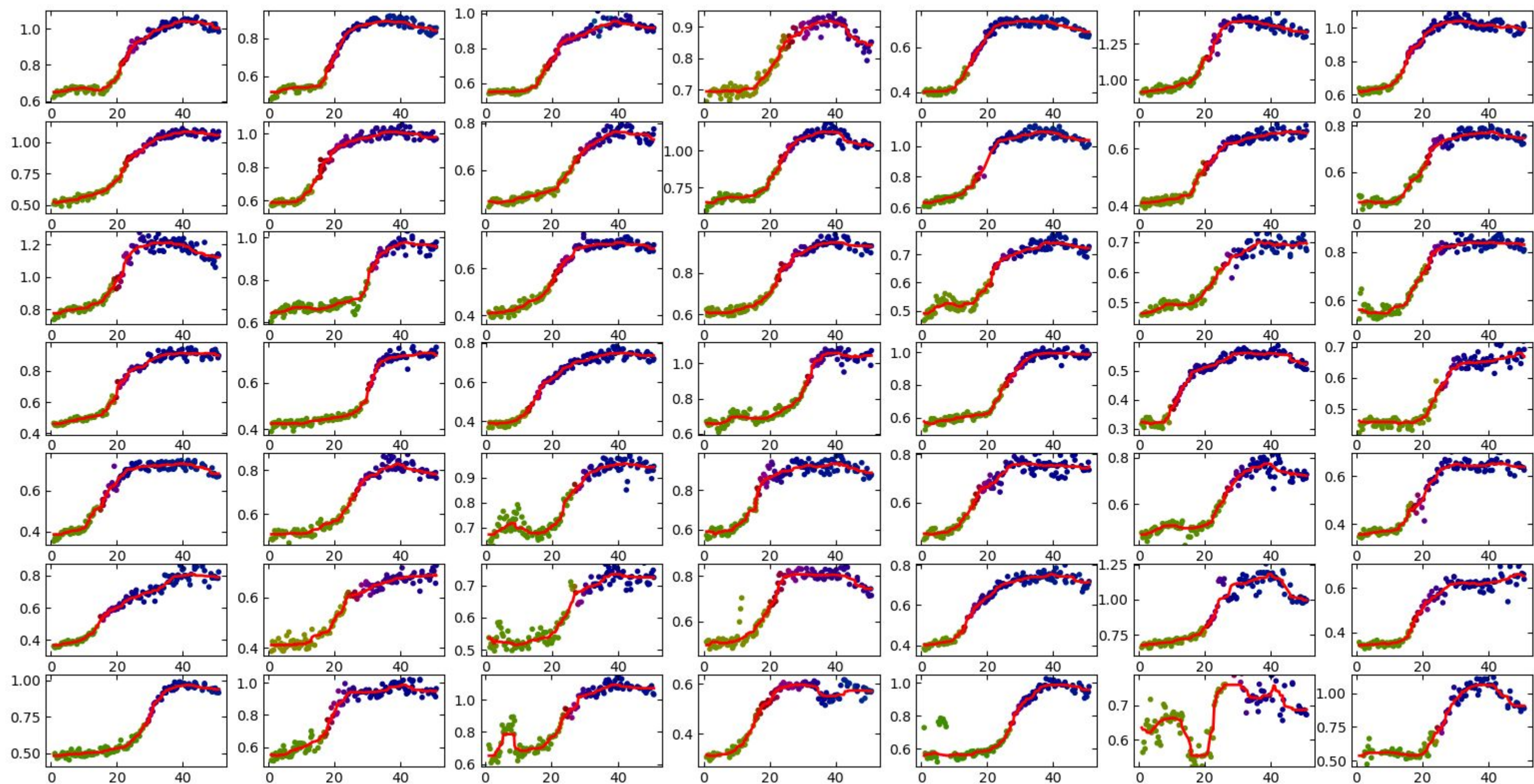**B**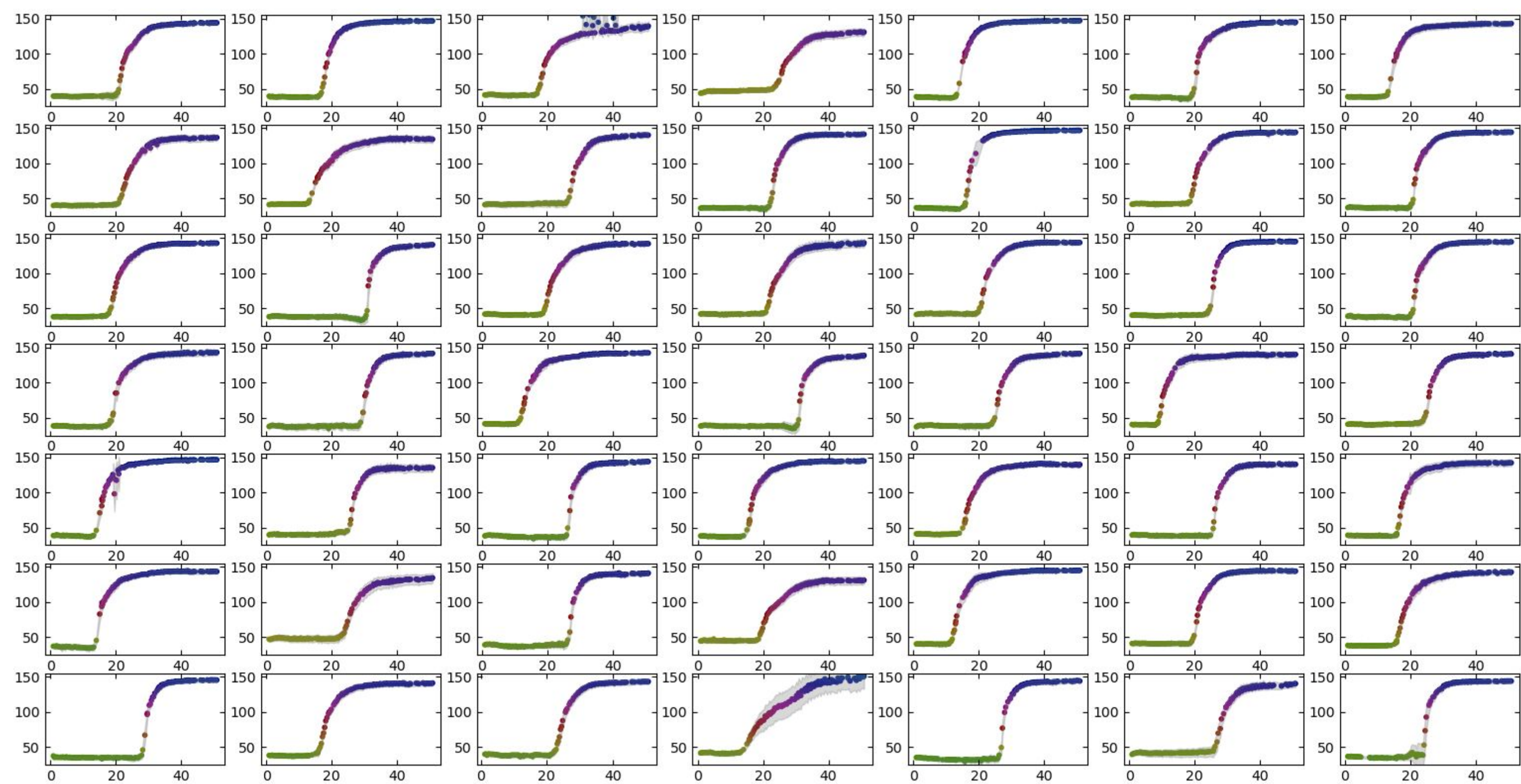

Supplement: Supplementary file 2 — Additional file 2: Growth and coloration kinetics of several individual grapevine berries. Repetition of the results shown in Fig. 7 for more berries. Each subplot displays the Volume (mL) (A) or Centred hue (deg) (B) measured over time (days) on an individual berry, after running the full image analysis pipeline on a time-series of 138 images, from 3 different camera views (120° difference) of the same grapevine bunch. All points are coloured using the corresponding average hue values. In A, the red curve corresponds to a 8-days moving median smoothing. In B, the grey area corresponds to the standard deviation of the centred hue value observed within the berry segmentation mask. [file 13007_2023_1125_MOESM2_ESM.pdf]

# 6 grapevine clusters

**A**

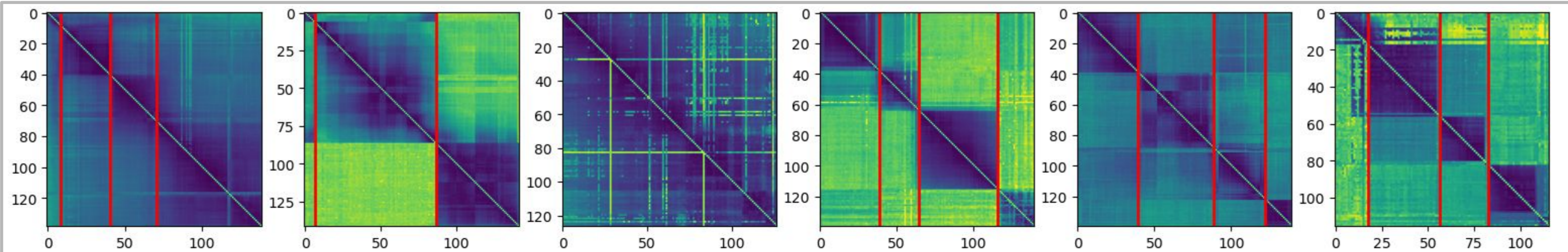

**B**

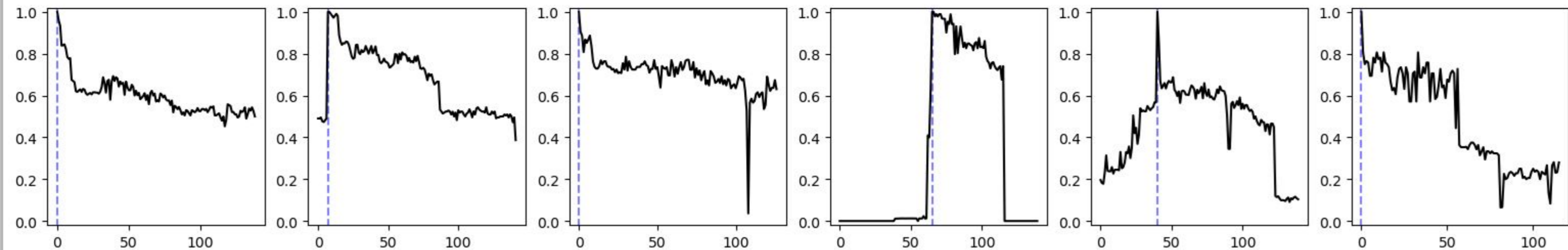

$$T_c = f(t)$$

Supplement: Supplementary file 4 — Additional file 4: Analysis of abrupt transitions in time-series of grapevine bunch images. A Heat map of the distance matrices obtained after tracking berries in time-series of 138 grapevine bunch images, for 6 different plants. Vertical red lines correspond to the empiric annotation of time-steps exhibiting abrupt transitions in these matrices. B Tracking coverage (${T}_{c}$Tc) over time obtained for these time-series. The dashed blue vertical line represents the time step ${t}_{root}$troot used to initialise the tracking. [file 13007_2023_1125_MOESM4_ESM.pdf]

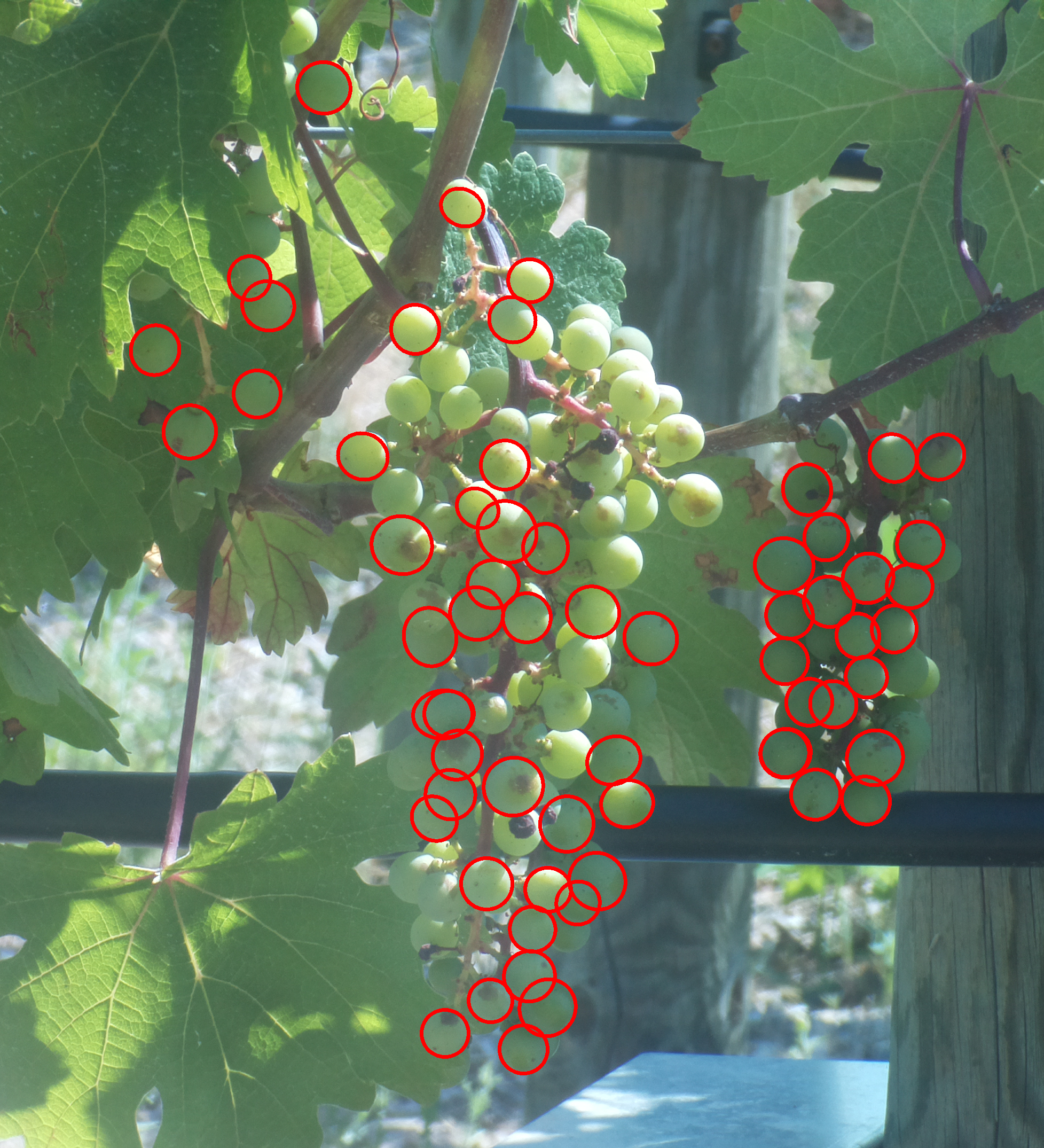

Supplement: Supplementary file 5 — Additional file 5: Detection and segmentation of berries in field conditions. Output of the berry detection and segmentation pipeline on an image of grapevine bunches taken in the field. This is a preliminary result. [file 13007_2023_1125_MOESM5_ESM.png]
