# Supplementary material for: Ripening dynamics revisited: an automated method to track the development of asynchronous berries on time-lapse images
Source: Plant Methods. 2023 Dec 14;19:146. doi: 10.1186/s13007-023-01125-8 (PMC10720176; doi:10.1186/s13007-023-01125-8)

ct = % of visible berry contours

A

	Pea-sized berry	Non-small berry ct = (50 ±10) %	Non-small berry ct < (50 ±10) %	Non-small berry ct > (50 ±10) %	Not a berry	Total
FP	3% 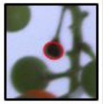	56% 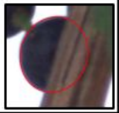	20% 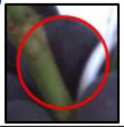	(always labeled)	23% 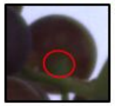 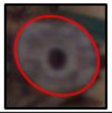	100% (n=64)
FN	27% 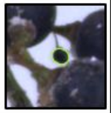	25% 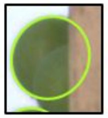	(never labeled)	47% 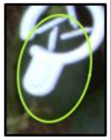	(never labeled)	100% (n=109)

B

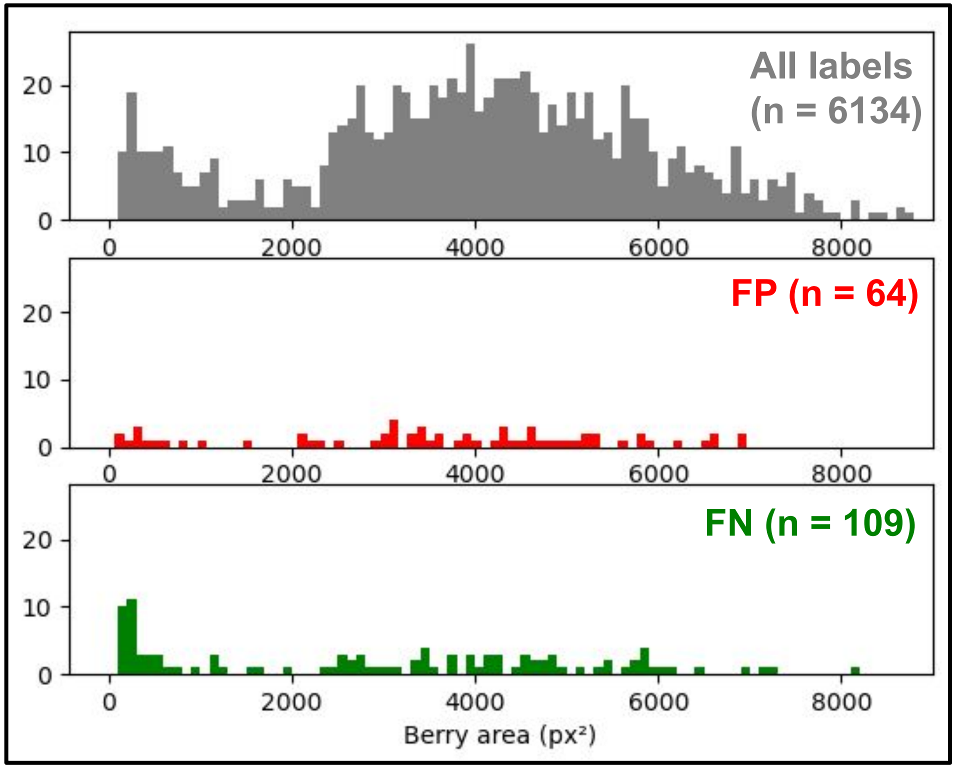

Supplement: Supplementary file 3 — Additional file 3: Analysis of berry detection errors in the test subset. Analysis of the False Positive (FP) and False Negative (FN) errors found when comparing berries detected by the pipeline to manually annotated berries, on the grapevine bunch images from the test subset. A Manual classification of detection errors as pea-sized berries, non-small (i.e. not pea-sized) berries, and non-berry objects. Non-small berries are further classified according to their percentage of visible contours (ct). B Distribution of detected berry sizes after segmentation, for all berries (top subplot), FP (middle subplot) and FN (bottom subplot). n: number of detected berries. [file 13007_2023_1125_MOESM3_ESM.pdf]
